# Which pharmacists are performing antimicrobial stewardship: A national survey and a call for collaborative efforts

**DOI:** 10.1017/ash.2021.245

**Published:** 2022-02-15

**Authors:** Brandon Dionne, Jamie L. Wagner, Daniel B. Chastain, Meagen Rosenthal, Monica V. Mahoney, Christopher M. Bland

**Affiliations:** 1 Department of Pharmacy and Health Systems Sciences, School of Pharmacy and Pharmaceutical Sciences, Northeastern University, Boston, Massachusetts; 2 Department of Pharmacy Practice, School of Pharmacy, University of Mississippi, Jackson, Mississippi; 3 Department of Clinical and Administrative Pharmacy, College of Pharmacy, University of Georgia, Albany, Georgia; 4 Department of Pharmacy Administration, School of Pharmacy, University of Mississippi, University, Mississippi; 5 Beth Israel Deaconess Medical Center, Boston, Massachusetts; 6 Department of Clinical and Administrative Pharmacy, College of Pharmacy, University of Georgia, Savannah, Georgia

## Abstract

**Objectives:**

To determine how pharmacists with formal antimicrobial stewardship program (ASP) responsibilities prioritize their time and pharmacists without formal antimicrobial stewardship program responsibilities contribute to ASP activities.

**Design:**

A nationwide survey.

**Respondents:**

Members of the American College of Clinical Pharmacy who subscribe to the following practice and research network e-mail listservs: infectious diseases, adult medicine, cardiology, critical care, hematology–oncology, immunology and transplantation, and pediatrics.

**Methods:**

A survey was distributed via listservs. Respondents were asked about their personal and institutional demographics and ASP activities.

**Results:**

In total, 245 pharmacists responded: 135 pharmacists with formal antimicrobial stewardship program responsibilities; 110 pharmacists without formal antimicrobial stewardship program responsibilities. Although most respondents had completed a general pharmacy residency (85%), only 20% had completed an infectious diseases (ID) specialty residency. Among pharmacists with formal antimicrobial stewardship program responsibilities, one-third had no formal training or certification in ID or ASP. Pharmacists without formal antimicrobial stewardship program responsibilities spent ∼12.5% of their time per week on ASP activities, whereas pharmacists with formal antimicrobial stewardship program responsibilities spent 28% of their time performing non-ASP activities. Pharmacists with formal antimicrobial stewardship program responsibilities were more likely than pharmacists without formal antimicrobial stewardship program responsibilities to perform antibiotic guideline development (*P* < .001), antibiotic-related education (*P* = .002), and direct notification of rapid diagnostic results (*P* = .018). Pharmacists with formal antimicrobial stewardship program responsibilities without formal ID training or certification spent less time on ASP activities and were more likely to perform lower-level interventions.

**Conclusions:**

Many ASP activities are being performed by pharmacists without formal ID training. To ensure the future success of ASPs, pharmacists with formal antimicrobial stewardship program responsibilities should have adequate training to meet more advanced metrics, and more pharmacists without formal antimicrobial stewardship program responsibilities should be included in basic interventions.

Antimicrobial stewardship programs (ASPs) have recently become a requirement for accreditation by The Joint Commission and a Condition of Participation for Medicare and Medicaid, which has led to a rapid expansion in the number and scope of ASPs. In 2019, 89% of hospitals reported that they met all 7 of the Centers for Disease Control and Prevention (CDC) Core Elements of a hospital ASP, up from just 41% in 2014.^1^ Although the Core Elements are an essential foundation, only one element, the “action” component, deals with the daily interventions utilized by an ASP. The Infectious Diseases Society of America (IDSA) and Society for Healthcare Epidemiology of America (SHEA) ASP guidelines provide a list of strategies that institutions can utilize, but these guidelines do not provide clear guidance on which to prioritize.^2^ Thus, the implementation of ASPs among institutions varies significantly, even those of similar size and setting.^3^

The growth in ASPs has also outpaced the number of infectious diseases (ID)–trained pharmacists, often requiring those without formal training through an ID residency or fellowship to take on ASP roles, sometimes in addition to their other duties.^3^ Although expertise in infectious diseases is not required by accrediting organizations to serve in the role of an ASP pharmacist, it is highly encouraged.^4^ Certification programs in antimicrobial stewardship exist to help bridge this gap in training, whereas board certification provides a pathway for pharmacists practicing antimicrobial stewardship to demonstrate expertise in infectious diseases. Unfortunately, data are lacking regarding how pharmacists who have formal ID training or certification compare to those without when fulfilling the role of an ASP pharmacist.

Although pharmacists with dedicated time for ASPs are most successful, those without formal responsibility for an ASP also contribute to antimicrobial stewardship efforts.^5–7^ Previous studies have evaluated the number of full-time equivalents (FTEs) and interventions used by ASP pharmacists; however, information on how these strategies are prioritized or how non-ASP pharmacists contribute is lacking. Because pharmacists often carry out the daily activities of an ASP, it is important to understand how these duties are currently performed. The objective of this survey was to determine how stewardship activities compare between pharmacists with and without formal responsibility for an ASP as well as how pharmacists with formal responsibility for an ASP compare in prioritization of stewardship strategies.

## Methods

A cross-sectional online survey was used to achieve the study objectives. Pharmacists who were members of the American College of Clinical Pharmacy and subscribe to the following Practice and Research Network (PRN) listservs were invited to participate in a survey on antimicrobial stewardship practices: adult medicine, cardiology, critical care, hematology–oncology, immunology and transplantation, infectious diseases, and/or pediatrics. The PRN listservs members were e-mailed in August 2019, and a follow-up reminder e-mail was sent 1 week later.

The main purposes of this survey were to characterize specific interventions and strategies used by ASP pharmacists, to quantify the time allocated to each of those activities, and to quantify and characterize how frontline pharmacists are performing ASP-related activities. Respondents were asked questions about their individual backgrounds, specifically years in practice since completing their training, any postgraduate training, certifications and credentials currently held, whether their job title or description included “infectious diseases” or “antimicrobial stewardship” or described antimicrobial stewardship activities, and whether they were formally responsible for an ASP regardless of their job title or description.

Questions surrounding the respondents’ practice sites included size and type of the institution, as well as the use of an electronic health record and computerized provider order entry. All respondents were also asked information about the status of the ASPs at their practice site, including duration of the formal program, physician or pharmacist involvement, methods to identify ASP interventions, ASP tools and practices, and personal ASP activities performed. Respondents were then divided into 2 groups based upon whether they reported having formal ASP responsibilities.

Those with formal responsibilities were also asked to comment on the percentage of time specifically dedicated to certain ASP activities, how they prioritize ASP responsibilities, and what metrics they use or report as part of the ASP. These ASP activities were categorized into basic stewardship (system-wide) interventions, intermediate stewardship (patient-specific) interventions, and advanced stewardship (diagnosis- and infection-specific) interventions according to the *National Quality Partners Playbook* classification of ASP responsibilities.^8^ Basic ASP activities included antibiotic spectrum recommendations, intravenous-to-oral (IV-to-PO) conversions, and pharmacokinetic dosing and adjustments. Intermediate metrics included antibiotic-related healthcare education, antibiotic duration of therapy recommendations, and antibiotic protocol, policy, and guideline development. Advanced metrics included recommendations to obtain antibiotic cultures or laboratory tests, provision of antibiotic-related healthcare education, and receipt of notification of rapid diagnostic results.

Pharmacists with formal ASP responsibilities were specifically asked to indicate their percentage of time spent on direct and indirect ASP activities. Pharmacists with formal ASP responsibilities were also asked how they would prioritize 6 different ASP activities: dose optimization, formulary restriction and preauthorization, guideline and clinical pathway development, IV-to-PO conversion, prospective audit with intervention and feedback, and streamlining or de-escalation of therapy.

We used SPSS version 26.0 software (IBM, Chicago, IL) for all data analyses. All nominal data were analyzed using the χ^2^ or the Fisher exact test, as appropriate, and all continuous variables were analyzed using the Student *t* test or Mann-Whitney *U* test, as appropriate. Statistical significance was reached if *P* values were <.05.

## Results

In total, 245 pharmacists responded to the survey, representing 44 states: 135 FR ASP pharmacists and 110 NFR ASP pharmacists. Of the 135 pharmacists with formal ASP responsibilities, 59 (44%) also had a formal job title that included “infectious diseases” or “antimicrobial stewardship,” and 90 (67%) participants received formal training in ID through an ID residency or fellowship, completed an ASP certificate program, and/or were currently board certified in ID. The median duration respondents had been practicing as a pharmacist since completing training was 10 years (IQR, 6–15). Additional respondent training and credentialing can be found in Table 1. Respondents were from a variety of institution types; however, similar ASP practices were performed at each site (Table 2).

**Table 1.** Respondent Characteristics

Variable	Total (n = 245), No. (%)	FR ASP Pharmacists (n = 135),No. (%)	NFR ASP Pharmacists (n = 110), No. (%)	*P* Value
**Additional postgraduate training**				
PGY1 pharmacy residency	209 (85.3)	112 (83)	97 (88.2)	.251
PGY2 pharmacy residency in infectious diseases	48 (19.6)	45 (33.3)	3 (2.7)	<.001
PGY2 pharmacy residency in other specialty	85 (34.7)	21 (15.6)	64 (58.2)	<.001
Ambulatory care	1 (0.4)	0 (0)	1 (0.9)	.449
Cardiology	8 (3.3)	2 (1.5)	6 (5.5)	.145
Critical care	32 (13.1)	10 (7.4)	22 (20)	.004
Drug information	1 (0.4)	0 (0)	1 (0.9)	.449
Emergency medicine	4 (3.6)	0 (0)	4 (3.6)	.039
Internal medicine	12 (4.9)	2 (1.5)	10 (9.1)	.007
Oncology	2 (0.8)	0 (0)	2 (1.8)	.201
Pediatrics	12 (4.9)	5 (3.7)	7 (6.4)	.383
Pharmacotherapy	4 (1.6)	0 (0)	4 (3.6)	.039
Psychiatry	1 (0.4)	1 (0.7)	0 (0)	1.000
Solid organ transplant	7 (2.9)	1 (0.7)	6 (5.5)	.048
Infectious diseases fellowship	10 (4.1)	7 (5.2)	3 (2.7)	.519
**Certification/Credentials**^**a**^				
BCPS	169 (69)	96 (71.1)	73 (66.4)	.424
BCIDP	40 (16.3)	40 (29.6)	0 (0)	<.001
BCCCP	42 (17.1)	13 (9.6)	29 (26.4)	.001
BCCP	10 (4.1)	3 (2.2)	7 (6.4)	.118
BCPPS	13 (5.3)	7 (5.2)	6 (5.5)	.925
BCOP	4 (1.6)	1 (0.7)	3 (2.7)	.329
CACP	1 (0.4)	1 (0.7)	0 (0)	1.000
AQ ID	15 (6.1)	13 (9.6)	2 (1.8)	.011
AQ Cardiology	1 (0.4)	1 (0.7)	0 (0)	1.000
AAHIVM HIV pharmacist	10 (4.1)	9 (6.7)	1 (0.9)	.025
Stewardship certificate program	38 (15.5)	34 (25.2)	4 (3.6)	<.001

Note. FR ASP, formal responsibilities for antimicrobial stewardship; NFR ASP, non-formal responsibilities for antimicrobial stewardship; PGY, postgraduate year; BCPS, board certified pharmacotherapy specialist; BCIDP, board certified infectious diseases pharmacist; BCCCP, board certified critical care pharmacist; BCCP, board certified cardiology pharmacist; BCPPS, board certified pediatric pharmacy specialist; BCOP, board certified oncology pharmacist; CACP, certified anticoagulation care provider; AQ ID, added qualifications in infectious diseases; AQ Cardiology, added qualifications in cardiology; AAHIVM, American Academy of HIV Medicine.

a >1 certification or credential could be selected.

**Table 2.** Institutional Characteristics and Antimicrobial Stewardship Practices

Variable	Total (n = 186), No. (%)	FR ASP Pharmacists (n = 110), No. (%)	NFR ASP Pharmacists (n = 76), No. (%)	*P* Value
**Institutional characteristics**				
Average size				
≤100 beds	8 (3.3)	8 (5.9)	0 (0)	.009
101–250 beds	44 (18)	34 (25.2)	10 (9.1)	.001
251–500 beds	77 (31.4)	29 (26.4)	48 (35.6)	.123
501–750 beds	58 (23.7)	27 (20)	31 (28.2)	.134
>750 beds	58 (23.7)	40 (36.4)	18 (13.3)	<.001
**Type of institution**^**a**^				
Academic medical center/University hospital	103 (42)	40 (29.6)	63 (57.3)	<.001
Community hospital	116 (47.3)	34 (30.9)	82 (60.7)	<.001
County hospital	11 (4.5)	8 (5.9)	3 (2.7)	.354
Federal facility (eg, VA, IHS, etc)	5 (2)	5 (3.7)	0 (0)	.066
Privately owned, for profit	12 (4.9)	3 (2.7)	9 (6.7)	.155
Privately owned, not for profit	25 (10.2)	13 (11.8)	12 (8.9)	.451
Specialty hospital	7 (2.9)	6 (4.4)	1 (0.9)	.133
Teaching hospital	86 (35.1)	36 (32.7)	50 (37)	.482
**Institutional ASP characteristics**				
Formalized ASP	242 (98.8)	135 (100)	107 (97.3)	.089
Years program established (IQR)	6 (4–10)	5 (3–8]	6 (5–10]	.038
Multiple sites covered by single ASP	112 (45.7)	63 (46.7)	49 (44.5)	.740
Pharmacist FTEs dedicated to ASP (IQR)	1 (1–2) (N=211)	1 (0.763–2) (N=116)	2 (1–3) (N=95)	<.001
1 RPh involved (n=215)	67 (31.2)	46/118 (39)	21/97 (21.6)	.006
2 RPh involved (n=215)	57 (26.5)	28/118 (23.7)	29/97 (29.9)	.308
≥3 RPh’s involved (n=215)	91 (42.3)	44/118 (37.3)	47/97 (48.5)	.099
**Institutional ASP tools and practices**				
Antimicrobial cycling	8 (4.3)	7 (6.4)	1 (1.3)	.144
Antimicrobial order forms	110 (59.1)	62 (56.4)	48 (63.2)	.354
β-lactam alternative dosing strategies	143 (76.9)	82 (74.5)	61 (80.3)	.363
Didactic education	124 (66.7)	71 (64.5)	53 (69.7)	.460
Hard stop dates for antibiotics not being given for surgical prophylaxis	71 (38.2)	38 (34.5)	33 (43.4)	.221
ID care bundles	140 (75.3)	90 (81.8)	50 (65.8)	.013
Indications for all antibiotic orders	130 (69.9)	74 (67.3)	56 (73.7)	.349
Non–culture-based fungal testing	110 (59.1)	66 (60)	44 (57.9)	.774
Penicillin allergy evaluation	89 (47.8)	50 (45.5)	39 (51.3)	.432
Pharmacokinetic monitoring of aminoglycosides and/or vancomycin	180 (96.8)	104 (94.5)	76 (100)	.083
Preauthorization	141 (75.8)	82 (74.5)	59 (77.6)	.629
Procalcitonin testing	129 (69.4)	78 (70.9)	51 (67.1)	.580
Prospective audit with feedback	151 (81.2)	92 (83.6)	59 (77.6)	.303
Rapid diagnostic testing on blood specimens	149 (80.1)	90 (81.8)	59 (77.6)	.482
Rapid viral testing for respiratory pathogens	140 (75.3)	85 (77.3)	55 (72.4)	.446

Note. FR ASP, formal responsibilities for antimicrobial stewardship; NFR ASP, nonformal responsibilities for antimicrobial stewardship; ASP, antimicrobial stewardship program; FTEs, full-time equivalents; ID, infectious diseases.

a >1 answer could be selected.

When respondents were asked which ASP activities they personally performed, >90% of their efforts focused on 4 main activities: antibiotic spectrum recommendations (98%; n = 182 of 186), antibiotic duration of therapy recommendations (96%; n = 178 of 186), IV-to-PO conversions (94%; n = 176 of 186), and pharmacokinetic dosing and adjustments (94%; n = 175 of 186). Notably, pharmacists without formal ASP responsibilities were more likely to rely upon team-based rounds as a primary method of identifying interventions than FR ASP pharmacists (33% vs 12%, *P* < .001). Pharmacists with formal ASP responsibilities performed a similar number of basic metrics to pharmacists without formal ASP responsibilities; however, they performed more intermediate and advanced metric interventions. Specific activities asked of the respondents can be found in Table 3. Pharmacists with formal ASP responsibilities were more likely to engage in antibiotic-related healthcare education and protocol development and to receive notification of rapid diagnostic results than pharmacists without formal ASP responsibilities.

**Table 3.** ASP Activities Performed by Respondent According to National Quality Partners Playbook Classification of ASP Responsibilities

Variable	Total (n = 186), No. (%)	FR ASP Pharmacists (n = 109), No. (%)	NFR ASP Pharmacists (n = 78), No. (%)	*P* Value
**Basic: systemwide interventions**				
Antibiotic duration of therapy recommendations^a^	178 (95.7)	104 (95.4)	75 (96.2)	1.000
Antibiotic protocol, policy, or guideline development^a^	139 (74.7)	94 (86.2)	45 (57.7)	<.001
Antibiotic spectrum recommendations	182 (97.8)	108 (99.1)	75 (96.2)	.310
Intravenous-to-oral conversions	176 (94.1)	101 (92.7)	75 (96.2)	.365
Pharmacokinetic dosing and adjustments	175 (93.6)	102 (93.6)	73 (93.6)	.997
**Intermediate: patient-specific interventions**				
Antibiotic duration of therapy recommendations^a^	178 (95.7)	104 (95.4)	75 (96.2)	1.000
Antibiotic protocol, policy, or guideline development^a^	139 (74.7)	94 (86.2)	45 (57.7)	<.001
Antibiotic-related healthcare education^a^	135 (72.2)	88 (80.7)	47 (60.3)	.002
**Advanced: diagnosis- and infection-specific interventions**				
Recommendations to obtain antibiotic cultures or laboratory tests	132 (71)	77 (70.6)	55 (70.5)	.985
Receive notification of rapid diagnostic results	91 (48.7)	61 (56)	30 (38.5)	.018
Antibiotic-related healthcare education^a^	135 (72.2)	88 (80.7)	47 (60.3)	.002

Note. FR ASP, formal responsibilities for antimicrobial stewardship; NFR ASP, nonformal responsibilities for antimicrobial stewardship.

a Classified in >1 category due to complexity of activity.

For pharmacists with formal ASP responsibilities, ∼60% of their time was spent performing direct ASP activities for all respondents. However, we identified significant differences between pharmacists with formal ASP responsibilities who had formal ID training or certification and those who did not. Namely, for pharmacists with formal ASP responsibilities, the percentage of time spent on direct compared to indirect ASP activities was significantly higher for those with formal ID training (70% of time) than those without formal ID training (35% of time; *P* < .001). A detailed breakdown of time is displayed in Table 4. Almost 50% of pharmacists with formal ASP responsibilities indicated that prospective audit with intervention and feedback was the most important ASP activity, followed closely by streamlining or de-escalation of therapy (44%) (Table 5). The least important ASP activity surveyed was IV-to-PO conversion (50%), with guideline and clinical pathway development close behind (36%).

**Table 4.** Percentage of Time Dedicated to the ASP in Pharmacists with Formal ASP Responsibilities

ASP Activity	Total (n = 118), Median (IQR)	Formal ID Training/ Certification (n = 76), Median (IQR)	No Formal ID Training/ Certification (n = 42), Median (IQR)	*P* Value
Pharmacist FTEs dedicated to ASP	1 (0.763–2)	1 (1–2)	1 (0.5–2)	.171
**Direct ASP activities**				
Review of restricted antimicrobials	4.5 (0–10)	5 (1–10)	0.5 (0–5)	.002
Antimicrobial de-escalation	15 (7–30)	20 (10–30)	10 (5–20)	<.001
Antimicrobial escalation/drug–pathogen mismatches	7.25 (5–10)	10 (5–15)	5 (2–10)	.022
Guideline and clinical pathway development and maintenance	5 (1–10)	5 (2–10)	5 (0–6.25)	.044
Parenteral to oral conversion	2.5 (0–5)	2 (0–5)	4.5 (1–10)	.023
Provider education	5 (1–5)	5 (1–8.75)	3.5 (1–5)	.396
Other ASP activities not listed	0 (0–10)	5 (0–10)	0 (0–5)	.001
Total direct ASP activities	60 (32.88–80)	70 (45–81.88)	35 (20–65)	<.001
**Indirect or non-ASP activities**				
Administration (e.g., committee work)	10 (5–15)	10 (5–20)	5 (1.75–10)	.001
Research	0 (0–5)	3.25 (1–8.75)	0 (0–5)	.023
Non-ASP activities (details not requested)	15 (0.75–51.25)	10 (0–24.5)	52.5 (15–71.25)	<.001
Total indirect or non-ASP activities	40 (20–67.13)	30 (12.13–55)	65 (35–80)	<.001

Note. ASP, antimicrobial stewardship; ID, infectious diseases; FTE, full-time equivalent

**Table 5.** Prioritizing ASP Responsibilities in Participants with Formal ASP Responsibilities

		Importance, Greatest (1) to Least (6), No. (%)					
Variable	Overall Ranking	1	2	3	4	5	6
Prospective audit with intervention and feedback	1	53 (48.2)	22 (20)	11 (10)	8 (7.3)	9 (8.2)	7 (6.4)
Streamlining or de-escalation of therapy	2	23 (20.9)	48 (43.6)	27 (24.5)	9 (8.2)	1 (0.9)	2 (1.8)
Dose optimization	3	12 (10.9)	24 (21.8)	32 (29.1)	28 (25.5)	10 (9.1)	4 (3.6)
Formulary restriction and preauthorization	4	10 (9.1)	10 (9.1)	17 (15.5)	25 (22.7)	28 (25.5)	20 (18.2)
Guideline and clinical pathway development	5	11 (10)	4 (3.6)	14 (12.7)	19 (17.3)	40 (36.4)	22 (20)
IV-to-PO conversion	6	1 (0.9)	2 (1.8)	9 (8.2)	21 (19.1)	22 (20)	55 (50)

Note. ASP, antimicrobial stewardship; IV-to-PO, intravenous-to-oral.

## Discussion

In our study, pharmacists with and without formal ASP responsibilities contributed to antimicrobial stewardship, but the amount of time expended and levels of complexity of interventions differed significantly between groups. Although pharmacists without formal ASP responsibilities spent an average of 0.125 FTE on antimicrobial stewardship, pharmacists with formal ASP responsibilities dedicated ∼0.6 FTE on antimicrobial stewardship clinical activities, even though the average program had 1 FTE allocated for an ASP pharmacist. An increase in pharmacist FTE has been associated with increased effectiveness of an ASP, and our findings suggest that this could potentially be accomplished by providing more protected time for pharmacists with formal ASP responsibilities to focus on clinical stewardship activities.^3^

We detected minimal differences between stewardship strategies utilized by the institutions of the 2 groups, suggesting that any differences between the groups in terms of ASP activities completed were due to individual rather than institutional practices. Pharmacists with formal ASP responsibilities were just as likely to personally complete most basic interventions, but they were more likely to accomplish advanced and intermediate stewardship metrics. This finding may be explained by the higher proportion of ID training or certification among pharmacists with formal ASP responsibilities, which allowed them to make more advanced interventions as well as by the fact that pharmacists without formal ASP responsibilities were more likely to identify interventions by rounding, which may have led to a higher proportion of basic interventions such as IV-to-PO interchange. By empowering pharmacists without formal ASP responsibilities to complete more basic and intermediate-level interventions, pharmacists with formal ASP responsibilities may be free to focus on the more complex activities that they feel are more important, such as prospective audit and feedback, which has previously been shown to be one of the most effective strategies.^3,9,10^ A teamwork approach of pharmacists both with and without formal ASP responsibilities involved in stewardship activities may be optimal to successfully meet all metrics, irrespective of complexity.

Although the guidelines recommend that ASPs include a pharmacist with ID training, currently only 126 PGY2 infectious diseases pharmacy residency programs are accredited by the American Society of Health Systems Pharmacists in the United States. That is, there is a shortage of programs to formally train all pharmacists who participate in stewardship activities.^2,11^ This shortage was evident in our results: only one-third of pharmacists with formal ASP responsibilities had completed a second postgraduate year in ID and only 5% had completed an ID fellowship. Even though most respondents had completed a 1-year postgraduate program, this training alone may not adequately prepare pharmacists to practice in antimicrobial stewardship, especially regarding intermediate and advanced interventions.^12^ Other ways to demonstrate competency include board certification or ASP certificate programs, but more than one-third of FR ASP respondents reported having neither.

Pharmacists with formal ASP responsibilities but without ID training or certification spent half as much time on direct ASP activities as those with training, despite having a similar number of pharmacist FTEs dedicated to the program. Possibly, the FTEs for the program may be split among multiple pharmacists who dedicated only part of their time to stewardship, but they still spent a higher proportion of their ASP time on basic activities such as IV-to-PO conversion and guideline development. There may also be pressure for ASPs, especially newly established programs, to achieve “low-hanging fruit” process-measure metrics to meet antimicrobial stewardship accreditation requirements, reduce costs, or to demonstrate their impact more easily.^13^ However, improvements in outcome measures achieved by more advanced interventions are becoming increasingly important for both reimbursement and accreditation.^14^ Additional education and training opportunities in ID and antimicrobial stewardship may allow these pharmacists to spend more time executing higher-level interventions. Multiple training or certificate programs are available, including a free online program from the Centers for Disease Control and Prevention (CDC); however, barriers to completion (e.g., awareness of these programs or the time or financial support to complete them) were not investigated in this study.

Our study had several limitations. A response rate could not be calculated because pharmacists could be members of >1 PRN, and membership also includes trainees and nonpracticing pharmacists, who were not eligible for the survey. The membership of ACCP includes clinical pharmacists from a variety of practice settings and backgrounds, which was reflected in the demographics of the respondents. The pharmacists without formal ASP responsibilities worked primarily in medium- or larger-sized teaching hospitals, and pharmacists without formal ASP responsibilities at smaller institutions may be less likely to attend rounds or be integrated with the primary teams to be able to make interventions as easily. Another limitation is that respondents only reported whether or not they performed a stewardship intervention without information on frequency or effectiveness. Previous studies have demonstrated that dedicated ASP pharmacists are more effective than decentralized pharmacists, so it would be important to monitor activities shifted to pharmacists without formal ASP responsibilities to ensure that they are being completed successfully.^6,7^ As ASPs continue to expand, it will be important to evaluate how pharmacists’ roles and training impact the effectiveness and efficiency of various strategies to determine how best to collaborate on the work of antimicrobial stewardship.

In conclusion, antimicrobial stewardship activities differ between pharmacists with and without formal ASP responsibilities. Each group utilizes different methods to identify potential antimicrobial stewardship interventions and practices to make recommendations. Due to the lack of adequate training programs and substantial amount of time pharmacists with formal ASP responsibilities spend on non-ASP, administrative, and research activities, many day-to-day clinical ASP activities are performed by pharmacists who lack antimicrobial stewardship certification, formal ID training, and/or do not have formal ASP job titles. Pharmacists responsible for ASP who lack formal antimicrobial stewardship or ID training should seek opportunities to obtain board certification in ID and/or ASP certification to ensure that pharmacists who complete more advanced stewardship activities are appropriately qualified while engaging frontline pharmacists without formal ASP responsibilities in basic and intermediate-level antimicrobial stewardship activities. Future research should explore barriers to ASP training for these pharmacists as well as for other clinicians.
